# Influence of Nanoclay Dispersion Methods on the Mechanical Behavior of E-Glass/Epoxy Nanocomposites

**DOI:** 10.3390/nano3030550

**Published:** 2013-08-28

**Authors:** Victor A. Agubra, Peter S. Owuor, Mahesh V. Hosur

**Affiliations:** 1 Materials Research and Education Center, Auburn University, 275 Wilmore Laboratories, Auburn, AL 36849, USA; 2 Center for Advanced Materials, Tuskegee University, Tuskegee, AL 36088, USA; E-Mails: powuor6138@mytu.tuskegee.edu (P.S.O.); hosur@mytu.tuskegee.edu (M.V.H.)

**Keywords:** nanoclays, nanocomposites, mechanical properties, dispersion methods

## Abstract

Common dispersion methods such as ultrasonic sonication, planetary centrifugal mixing and magnetic dispersion have been used extensively to achieve moderate exfoliation of nanoparticles in polymer matrix. In this study, the effect of adding three roll milling to these three dispersion methods for nanoclay dispersion into epoxy matrix was investigated. A combination of each of these mixing methods with three roll milling showed varying results relative to the unmodified polymer laminate. A significant exfoliation of the nanoparticles in the polymer structure was obtained by dispersing the nanoclay combining three roll milling to magnetic and planetary centrifugal mixing methods. This exfoliation promoted a stronger interfacial bond between the matrix and the fiber, which increased the final properties of the E-glass/epoxy nanocomposite. However, a combination of ultrasound sonication and three roll milling on the other hand, resulted in poor clay exfoliation; the sonication process degraded the polymer network, which adversely affected the nanocomposite final properties relative to the unmodified E-glass/epoxy polymer.

## 1. Introduction

Incorporation of nanoparticles to thermosets and thermoplastics polymers have been shown to greatly increase the final nanocomposite properties. A key component to achieving these improvements in properties lies in the proper dispersion of the nanoparticles in the polymer matrix. Some of the most commonly used methods for dispersing nanoparticles in polymers include: mechanical mixing, magnetic stirring, and sonication. It has been reported by many researchers that poorly dispersed nanoparticles could degrade the mechanical properties of polymers [1,2,3,4]. Depending on the mixing technique used, conventional nanoclay reinforced composites can take the form of phase-separated microcomposite, intercalated nanocomposites, or exfoliated nanocomposites [5]. Phase-separated microcomposites offer little improvement in material properties while exfoliated nanocomposites are reported to show the greatest interfacial interaction and phase homogeneity [6,7]. Therefore, the degree of exfoliation is an important parameter to evaluate the physical properties of polymer-based nanocomposites [8]. Numerous studies have extensively explored many of these mixing techniques to disperse nanoparticles in polymers with the aim of achieving full exfoliation. Among these includes *in-situ* intercalative polymerization approach, which was first successfully used in the manufacture of nylon-montmorillonite thermoplastics based nanocomposites [9]. Other techniques include melt intercalation method, and methods that use conventional shear devices such as sonicators, extruders, three-roll milling, or ball milling [5,10,11,12]. Regardless of the type of mixing technique employed, full exfoliation, which is a state in which all layers of the nanoclay are separated from all tactoids of clay [13], is difficult to achieve. This can be attributed to the high intrinsic viscosity of the resin, large lateral dimensions of the silicate layers, and strong tendency of clay platelet to agglomerate [14]. Generally, a weak interphase between the fiber and the epoxy results in matrix/fiber debonding, which eventually lead to the deterioration of the final properties of the nanocomposite. Montmorillonite nanoclay, a commonly used nanofiller, has high aspect ratio and a large interface, therefore its incorporation in the polymer matrix generates a large surface area [15] for the polymer/filler interaction to provide the needed reinforcement [16]. The benefit of the clay as a filler depends on the amount added to the polymer matrix [17]. Large amounts of montmorillonite clay in the polymer tend to negate its usefulness as a filler. This is attributed to the fact that, large amount of the clay tends to agglomerate, increase the viscosity of resin, decrease the reaction enthalpy, and decrease fiber/epoxy interface interaction [18]. Therefore large clay content tends to deteriorate the final properties of the nanocomposite. It is apparent that mixing or dispersion methods influence to a large degree the state of the clay (exfoliation) in the polymer matrix. From a thermodynamic perspective, clay exfoliation occurs due to the monomeric polar epoxy resin being attracted by the high surface energy of the clay diffusing into the interclay galleries until an equilibrium state is established [19]. Another definition of exfoliation in nanocomposites is that state when the clay layer spacing increases to the point where the attraction between the clay particles no longer exist [20]. The many different dispersion techniques lead to varying degrees of exfoliation. Dispersion methods such as sonication, magnetic stirring, and thinky mixing each achieve reasonable exfoliation of the nanoclay particles in the polymer. However, three roll milling is an effective dispersion method that disentangles the nanoclay particles and increases the clay galleries that enable the epoxy molecules to penetrate [21]. The three roll milling effectively reduces the inefficiencies of the individual mixing methods. This paper therefore seeks to investigate the effect of combining three roll milling with three different mixing methods- ultrasound sonication, thinky and magnetic mixing on nanoclay exfoliation and its influence on the mechanical and viscoelastic properties of E-glass/epoxy nanocomposite.

## 2. Results and Discussion

### 2.1. Mechanical and Viscoelastic Properties

The flexural, compressive and the viscoelastic properties of the glass/epoxy nanocomposite laminates are summarized in Table 1 Table 2, Table 3 respectively, for the various clay dispersion techniques. For the method 1 dispersion procedure, the E-glass/epoxy nanocomposite loss was an average of 12% of its maximum flexural stress for the 1 wt.% and 2 wt.% relative to the unmodified glass/epoxy polymer. The 3 wt.% laminates however, loss 22% of its flexural stress. This large degradation of the flexural properties was attributed to the ultrasound sonication process. The ultrasound sonication process tends to not only break down the large clay clusters but does not influence exfoliation of the nanoclay in the polymer resin. In essence, sonication does not influence the interplanar distance between the nanoclay platelet [5]. Although the shear force on resin/nanoclay mixture from the subsequent three roll milling increased the basal spacing of the clay platelet in the epoxy, the heat generated at the sonicators horn tend to affect the polymer network. This degradation of flexural properties from ultrasonic mixing of nanoclay particles agrees with the findings of Chun-ki Lam *et al*. [16]. Their study reported a degradation of mechanical properties of the composite polymer after exceeding the optimum sonication time of 10 min. Secondly, the relative high loss recorded for the 3 wt.% nanoclay samples was attributed to low nanoclay exfoliation, particle agglomeration, and the high viscosity of the resin-nanoclay mixture of the 3 wt.% of nanoclay. The high viscosity posed a difficulty in the resin infusing process which resulted in poor fiber wetting. There was, however, improvement in both the compressive stress and the viscoelastic properties of the E-glass/epoxy nanocomposites using method 1 as a means of dispersing the nanoclay particles in the polymer resin. In fact, adding three roll milling to the magnetic stirring resulted in an increased in flexural properties of the post cured E-glass/epoxy nanocomposite polymer compared to what was reported in our previous study [22]. Generally, a stronger interfacial bond is created by the nanoclay in the polymer, improving the viscoelastic properties of the composite E-glass/epoxy nanocomposite. These strong interfacial bonds restrict molecular mobility of the polymer chain, and depended largely on the degree of exfoliation in the polymer. Compressive properties are generally affected by matrix softening, delamination, and cracking. In addition, compressive failure mechanism in fiber reinforced composites is usually in the form of fiber microbuckling, which depended to a lesser extent on the existence of strong interfacial bonds between the fiber/matrix but more on the local instability of fibers embedded in the matrix [1].

**Table 1:** Flexural properties.

Mixing method	Sample	Max stress (MPa)	% increment	Max strain	% increment	Modulus (GPa)	% increment
Sonication + three roll milling	Neat epoxy	914.57 ± 22		2.97 ± 0.1		29.39 ± 1.0	
	1% wt.%	797.73 ± 38	−12.78	2.79 ± 0.2	−5.98	28.69 ± 0.8	−2.37
	2% wt.%	799.81 ± 18	−12.55	2.80 ± 0.2	−5.72	27.74 ± 2.0	−5.60
	3% wt.%	709.68 ± 31	−22.40	2.58 ± 0.1	−13.30	26.33 ± 3.1	−10.40
Thinky + three roll milling	1% wt.%	919.50 ± 17	0.54	3.08 ± 0.1	4.05	29.44 ± 1.1	0.20
	2% wt.%	926.19 ± 35	1.27	2.81 ± 0.3	2.36	30.21 ± 1.2	2.81
	3% wt.%	806.41 ± 23	−11.83	2.83 ± 0.3	−4.39	26.54 ± 2.2	−9.69
Magnetic stirring + three roll milling	1% wt.%	922.64 ± 15	0.88	3.10 ± 0.1	4.73	29.98 ± 0.8	2.02
	2% wt.%	1033.74 ± 12	13.03	2.94 ± 0.1	0.33	32.08 ± 0.6	9.17
	3% wt.%	919.38 ± 14	0.53	3.02 ± 0.1	2.03	30.42 ± 0.6	3.51

**Table 2:** Compressive properties.

Mixing method	Sample	Max stress (MPa)	% increment	Max strain	% increment	Modulus (GPa)	% increment
Sonication + three roll milling	Neat epoxy	414.02 ± 14		0.10 ± 0.01		11.2 ± 1.2	
	1% wt.%	427.93 ± 10	3.36	0.11 ± 0.01	4.30	12.3 ± 2.01	9.82
	2% wt.%	431.14 ± 13	4.14	0.11 ± 0.02	6.78	11.88 ± 1.3	6.07
	3% wt.%	384.13 ± 28	−7.22	0.10 ± 0.01	−3.31	12.13 ± 1.5	8.30
Thinky + three roll milling	1% wt.%	430.85 ± 18	4.07	0.11 ± 0.01	4.74	12.7 ± 2.1	13.39
	2% wt.%	434.91 ± 21	5.05	0.11 ± 0.01	7.40	11.73 ± 1.8	4.73
	3% wt.%	386.23 ± 25	−6.71	0.10 ± 0.02	0.26	11.4 ± 1.1	1.79
Magnetic stirring + three roll milling	1% wt.%	436.55 ± 18	5.44	0.11 ± 0.01	6.61	11.73 ± 1.2	4.73
	2% wt.%	493.42 ± 16	19.18	0.11 ± 0.01	10.37	12.08 ± 1.2	7.86
	3% wt.%	475.18 ± 21	14.77	0.10 ± 0.02	1.67	12.73 ± 0.8	5.38

**Table 3:** Viscoelastic properties.

Mixing method	Sample	Storage modulus (GPa)	% increment	Lost modulus (GPa)	% increment	Tan delta	% increment	Tg (oc)	% increment
Sonication + three roll milling	Neat epoxy/epoxy	22.11 ± 1.05		2.98 ± 0.1		0.345 ± 0.1		116.31 ± 0.9	
	1 wt.%	24.79 ± 4.74	10.80	3.48 ± 0.2	14.19	0.36 ± 0.1	3.81	106.04 ± 1.02	−9.69
	2 wt.%	28.76 ± 0.98	23.11	2.82 ± 0.1	−5.67	0.33 ± 0.2	−5.51	104.74 ± 1.07	−11.04
	3 wt.%	22.50 ± 3.22	1.70	2.61 ± 0.3	−14.16	0.31 ± 0.1	−12.03	112.64 ± 1.5	−3.26
Thinky + three roll milling	1 wt.%	26.56 ± 2.02	16.75	3.61 ± 0.2	17.28	0.36 ± 0.1	2.82	116.33 ± 0.6	0.01
	2 wt.%	30.43 ± 0.96	27.32	4.42 ± 0.1	32.45	0.37 ± 0.1	7.26	111.18 ± 4.44	−4.61
	3 wt.%	23.95 ± 2.48	7.66	3.21 ± 0.4	6.96	0.35 ± 0.1	0.96	116.67 ± 1.8	0.31
Magnetic stirring + three roll milling	1 wt.%	47.47 ± 3.52	53.42	6.42 ± 0.1	53.55	0.37 ± 0.2	6.09	116.84 ± 0.2	0.45
	2 wt.%	39.92 ± 7.35	44.60	5.72 ± 0.1	47.84	0.38 ± 0.1	8.50	114.18 ± 0.32	−1.87
	3 wt.%	37.64 ± 1.36	41.25	5.83 ± 0.3	48.86	0.36 ± 0.1	3.72	120.98 ± 0.4	3.86

The results from the second dispersion procedure (method 2) showed moderate increases in the flexural, compressive and viscoelastic properties for the E-glass/epoxy nanocomposites. The centrifugal force initiated particle-particle collision and the high pressure environment effectively broke down the electrostatic binding force holding the clay bundles together. This increased the interclay galleries for the epoxy molecules to diffuse into. The vacuum environment further reduced the incidence of bubbles and void content in the resin/clay mixture. The addition of the three roll milling process further enhanced exfoliation of the nanoclay particles. The loss in both flexural and viscoelastic properties for the 3 wt.% was attributed to difficulty in dispersion resulting from particles agglomeration and smaller d-spacing for the clay galleries as shown in Table 4. The high clay content also increased the viscosity of the resin and posed a challenge for infusing the resin-nanoclay mixture into the E-glass fiber mat. This in fact, affected uniform wetting of the E-glass fiber.

**Table 4:** Extracted X-ray diffraction parameters for the nanocomposites polymer laminates.

Sample	2θ	d-sapcing (Å)	RI
Pure nanoclay	16.3	4.50	-
2 wt.% magnetic + three roll milling	13.7	4.80	6.7
2 wt.% Sonication + three roll milling	14.2	4.70	4.4
3 wt.% magnetic + three roll milling	12.4	4.60	2.5
3 wt.% Sonication + three roll milling	12.7	4.55	1.1

**Figure 1:**
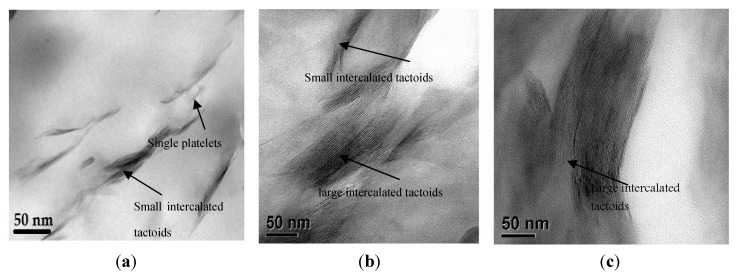
Transmission electron microscopy (TEM) micrographs of dispersied nanoclay particles for (**a**) 2 wt.% magnetic + 3 roll milling; (**b**) 3 wt.% magnetic + 3 roll milling; (**c**) 3 wt.% sonication + 3 roll milling.

**Figure 2:**
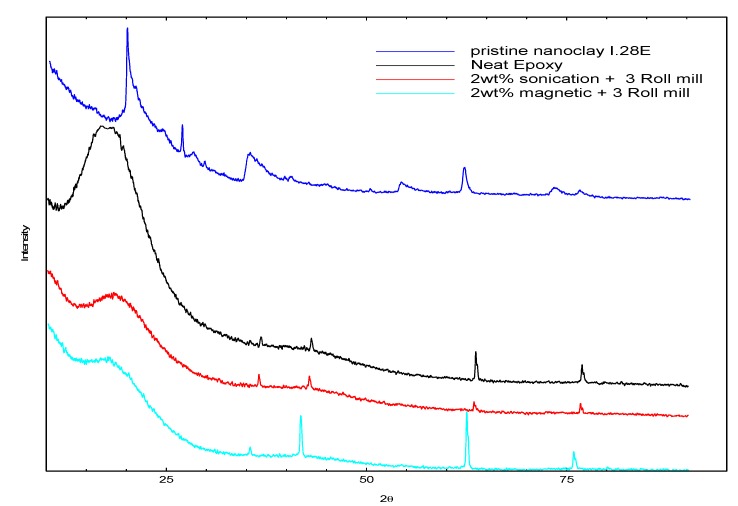
X-ray diffraction for pristine nanoclay, neat epoxy and nanocomposite polymer of 2 wt.% for sonication and magnetic mixing methods.

There was a general increase in the flexural, compressive and viscoelastic properties when the clay was dispersed using the third method of dispersion (method 3). These increases for flexural stress were substantial for the 2 wt.%, recording a 13% increase relative to the unmodified E-glass/epoxy composite. While that for the 1 wt.% and 3 wt.% recorded less than 1% increases in flexural stress. The combination of the magnetic stirring force and the shear force from the rollers of the calendar mixing method successfully exfoliate clay in the polymer resin, as evident in the morphology (Figure 1) and X-ray diffraction analysis (Figure 2). Therefore the significant increase in the flexural, compressive and viscoelastic properties for the magnetic and three roll milling can be attributed to exfoliation of the nanoclay particles in the polymer using this combination of dispersion. To investigate this further, X-ray diffraction and transmission electron microscopy (TEM) analysis were carried out on the 2 wt.% and 3 wt.%. These two weight percentages of clay recorded varying compressive and flexural properties using sonication and magnetic followed by three roll milling. Although X-ray diffraction analysis may indicate clay layer spacing in the polymer, it is not an ideal method for showing the spatial distribution of the silicate layers or any structural inhomogeneity in the E-glass/epoxy nanocomposite. Nonetheless, X-ray diffraction studies complemented by TEM have been used extensively to study the state of exfoliation of nanoparticles in polymers [5,9,13,19,21]. 

### 2.2. Morphology and Structural Analysis

**Figure 3:**
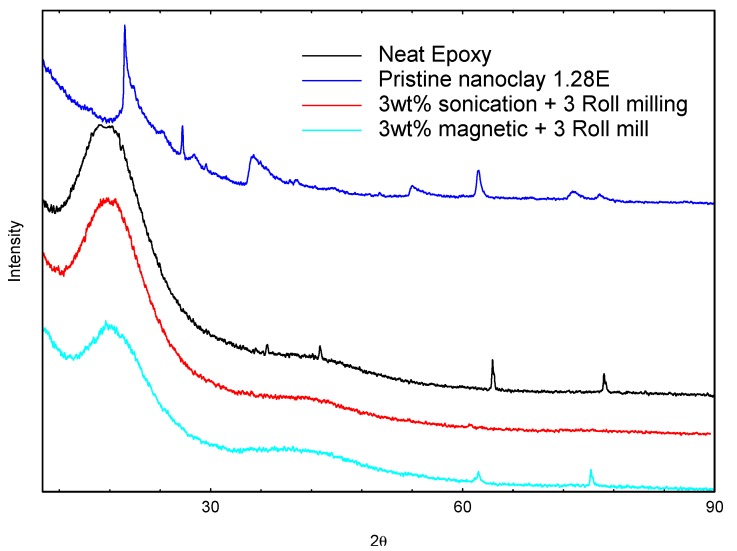
X-ray diffraction for pristine nanoclay, neat epoxy and nanocomposite polymer of 3 wt.% for sonication and magnetic mixing methods.

Morphology and structural analysis were undertaken on the 3 wt.% and 2 wt.% for the dispersion (methods 1 and 3). These two categories recorded varied results; a loss of 22% for the 3 wt.% using (method 1) and a gain of 13% for the 2 wt.% for (method 3) relative to the baseline. The samples were separately cured in a mold without the E-glass reinforcement. The X-ray diffraction spectra for the 2 wt.% and the 3 wt.% are shown in Figure 2, Figure 3 respectively. The diffraction pattern for the pristine montmorillonite nanoclay I.28E shows a strong (001) basal peak at 2θ~16° indicating a basal spacing of the nanoclay galleries of 4.5 Å, with a second and third peaks appearing at ~27° and 67° corresponding to a lattice d-spacing of 3.2 Å and 1.5 Å respectively. As shown in Figure 2, the neat epoxy showed a broad amorphous peak at 2θ~13° and four other additional peaks at 2θ~35°, 42°, 62° and 74°, it was also observed that the 2 wt.% nanocomposite polymer had all the peak pattern corresponding to the neat epoxy. However, there was a significant reduction in the amorphous peak intensity, an indication of the increase in crystallinity of the polymer laminate with the incorporation of the nanoparticles into the epoxy.

The X-ray diffraction for the two mixing sequence for the 2 wt.% in Figure 2, showed same peak pattern as in the neat polymer. Nevertheless, there was a slight shift in peak position relative to the neat polymer peak patterns, an indication of structural change in the polymer network. The clay galleries (d-spacing) for the two mixing sequence showed little variation and a difference of approximately 0.3 Å compared to the lattice spacing existing in the pristine clay as shown in Table 4. Increases in the gallery spacing allow the epoxy molecules to penetrate between the clay sheets to promote a strong intercalation of the nanoclay platelets in the epoxy. The absence of the clay peaks in the nanocomposites polymer samples can be attributed to the higher basal spacing and small amount of the clay platelets in the epoxy. The orientation or the intercalation of the epoxy molecules into the clay galleries was determined from X-ray diffraction spectra using the relative intercalation index (RI) derived from the mathematical relation [23].

*RI* = [(*d* − *d*_0_)/*d*_0_] × 100 (1)

where *d*_0_ is the lattice spacing of the pristine clay particles and *d* is the lattice spacing of the composite polymer (nanoclay + epoxy). These RI results are summarized in Table 4. The magnetic stirring followed by three roll milling for the 2 wt.% polymer showed a significant difference in relative intercalation index values. This improvement of the relative intercalation for this mixing method over the sonication followed by three roll milling accounted for the 13% increase in the flexural stress. The homogeneity of the clay particles in the epoxy was confirmed through a complementary TEM analysis of the samples as shown in Figure 1a. In the TEM micrographs, single clay platelets as well as small intercalated tactoids were randomly distributed throughout the polymer matrix. Clearly, these results showed that the dispersion sequence of the magnetic stirring followed by three roll milling resulted in better exfoliation of the nanoclay particles in the polymer matrix.

The X-ray diffraction spectra for the 3 wt.% polymer samples for the two different mixing sequences are presented in Figure 3. The X-ray patterns for the pristine clay and neat epoxy polymer are repeated in the plot for easy comparison. There was a reduction in the amorphous peak similar to that observed in the 2 wt.% nanocomposite polymer X-ray patterns. For the 3 wt.% magnetic stirring followed by three roll milling sample, only two peaks were observed corresponding to the last peak of the neat epoxy polymer observed at 2θ~62° and 74°. In addition, there was a second amorphous peak observed at 2θ~42°. A reduction in the peak intensity and slight shift in peak position for the last two peaks characterized the peak patterns for the 3 wt.% magnetic stirring followed by three roll milling nanocomposite sample. The higher nanoclay content in the polymer resulted in the relative decrease in the peak intensity which affected the degree of coherent stacking of the silicate layers in the polymer. This led to the formation of heterogeneous microstructure of the nanoclay in the polymer. Further analysis of the relative intercalated index showed low value of 2.5 and difference of 0.1 Å in the d-spacing relative to the pristine montmorillonite nanoclay. On the other hand, the X-ray spectra for the 3 wt.% sonication followed by three roll milling showed no other peak except the amorphous peak. The absence of peaks attributed to the neat polymer from the 3 wt.% sonication followed by three roll milling samples cannot readily be explained. However, we believe that the heat dissipated from sonication process might have adversely affected the polymer network. The TEM analysis on these samples is shown in Figure 1b,c. The morphology for the magnetic stirring followed by three roll milling showed the presence of large and small intercalated tactoids of clay platelets distributed heterogeneously in the polymer matrix. On the other hand, the morphology of the clay particles in the polymer matrix for the 3 wt.% sonication followed by three roll milling is characterized by large agglomerates clay intercalation tactoids in the polymer matrix. The TEM micrographs of the clay morphologies in the polymer clearly confirmed the poor clay exfoliation in the polymer matrix for the high nanoclay content due to particle agglomeration and high viscosity of the resin-nanoclay mixture.

**Figure 4:**
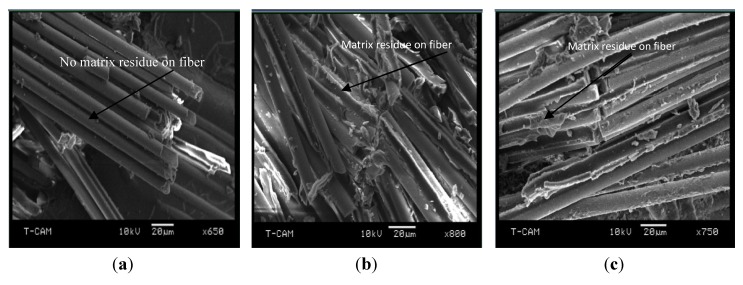
SEM micrographs of factured surface for the nanocomposite laminates (**a**) Neat E-glass/epoxy; (**b**) 2 wt.% magnetic + 3 roll milling; (**c**) 3 wt.% Sonication + 3 roll milling.

The fractographs presented in Figure 4 did not show any differing failure mechanisms among the various dispersion methods. The only distinctive feature is the residue of matrix attached to the fiber after breakage among all the nanocomposite polymer samples, which was conspicuously missing in the unmodified E-glass/epoxy polymer sample. This is indicative of the formation of an interfacial bond between fiber and matrix created by the clay sheets. Common failure mechanisms observed across the board included fiber-matrix debonding, fiber pullouts, fiber breakage, and matrix cracking in the direction of the applied load.

## 3. Experimental Section

### 3.1. Material Selection and Fabrication of Specimen

Materials used for this investigation included: unidirectional E-glass fibers obtained from Fiberglast, a two-part epoxy (SC-1) polymer resin from Applied Poleramic Inc. and montmorillonite nanoclay I.28E from Sigma Aldrich. The montmorillonite nanoclay (I.28E) is a 2-layered plate-like structure of 1 nm thickness and a lateral dimension of several microns giving the clay its high aspect ratio. The I.28E montmorillonite clay has mean particle size of 15–20 µm, a bulk density of 250–300 kg/m^3^ and the *d*_001_ diffraction of about 4–8 Å. The clay was treated with Onium cations of amine functionality to improve the compatibility of the hydrophilic clay with that of the hydrophobic epoxy. This treatment was also intended to lower the surface energy of the inorganic clay to enhance intercalation. To ensure that no moisture was entrapped in the nanoclay, a measured amount of nanoclay was pre-heated at 60 °C for 2 h. Using the recommended mixing ratio of 100:22 for the resin and hardener, the appropriate weight percentage of nanoclay was added to the epoxy resin—Part A and manually stirred to obtain a partial uniform paste. The nanoclay was then dispersed in the epoxy resin using three different mixing combinations; ultrasonic sonication followed by three roll milling, thinky mixing followed by three roll milling, and magnetic stirring followed by three roll milling. The procedures used in these mixing combinations are outlined in the following sections.

### 3.2. Method 1: Ultrasonic Sonication Followed by Three Roll Milling

**Figure 5:**
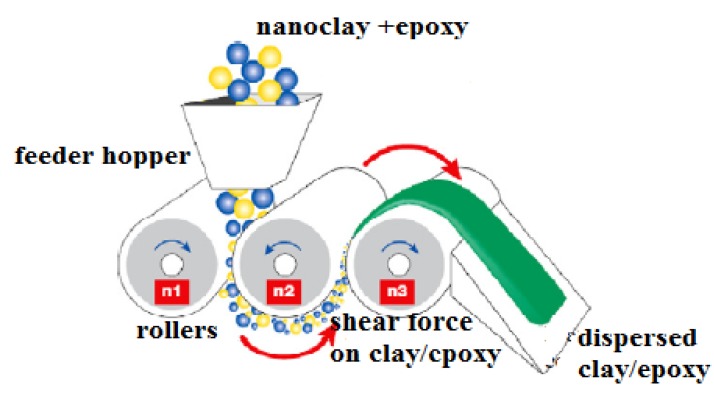
Three roll Milling process [5].

Sonication of the resin and nanoclay was done over a coolant bath for 1 h. For the sonication process a pulse rate of 50:25 and amplitude of 25% were used a low sonication horn energy of 25% was chosen to minimized heat generation which tends to degrade the polymer. In the ultrasonic process, the ultra sound propagates attenuated waves through the clay particle molecules “peeling off” the individual nanoparticles from the clay bundles thus opening the clay galleries (interlayer spacing) to the epoxy resin [13]. The clay-resin mixture was further processed using the calendar process commonly referred to as three roll milling. The three roll milling machine generally consist of three adjacent cylindrical rollers each running at different velocities. The resin was fed into the rollers as depicted in Figure 5. As the resin passes between these rollers, it was subjected to shear force which disentangled the nanoclay particles and distribute the dispersed clay into the resin. The shear force applied to the resin and clay particles overcame the cumulative attractive force binding the clay platelets. Three roll milling was carried out using different passes with gap setting between the rollers from 35 µm down to 15 µm, in three passes.

### 3.3. Method 2: Thinky Mixing (Planetary Centrifugal Vacuum Mixer) Followed by Three Roll Milling

The thinky mixing process involves centrifugal force rotation of the nanoclay-epoxy mixture under vacuum pressure. This initiated particle-particle collision thus breaking the electrostatic force holding the clay bundles together to disperse the nanoclay. The partial uniform paste of the resin and nanoclay was thinky mixed for 1 h at pressure of 30 psi and speed of 1200 rpm. A further mixing of three roll milling was then carried out using different passes with gap setting between the rollers from 35 microns down to 15 microns.

### 3.4. Method 3: Magnetic Stirring Followed by Three Roll Milling

The partial uniform paste of the resin and nanoclay was stirred using magnetic stirring for 24 h (longer period was used because of the slow rate). The vortex effect from the stirring magnet created a particle-particle collision and collision with the magnetic bar. This process breaks down the electrostatic force that held the particle agglomerates to produce a partial dispersion of the clay in the epoxy resin. A final mixing using the three roll mill was then carried out using different passes with gap setting between the rollers from 35 microns down to 15 microns, in three passing in steps of 15 microns.

### 3.5. Composite Fabrication

An appropriate amount of hardener (Part B) of the resin was added to the three categories of the dispersed clay/resin mixture and mechanically stirred. The polymer/nanoclay mixture was then degassed to remove air bubbles trapped in the resin. The vacuum assisted resin transfer method (VARTM) was employed to infuse the polymer/nanoclay mixture into a prearranged (20 layers) unidirectional E-glass fibers and allowed to cure at ambient temperature. The same procedure was used to fabricate composite laminates of 0 wt.% (unmodified), 1 wt.%, 2 wt.%, and 3 wt.% nanoclay loading. The composite laminates were then post cured at 90 °C for 3 h. Post curing the polymer nanocomposite at a higher temperature to enhance the intercalation of the nanoclay particles in the epoxy has been reported in [5].

### 3.6. Mechanical and Viscoelastic Testing

Sample size of five was prepared according to ASTM D7137-12 for the quasi-static compression test using a servo-hydraulically controlled material testing system (MTS) machine. The test was carried out in displacement control mode with cross-head speed of 1.27 mm/min. The load-deflection data recorded by the data acquisition system was converted to stress-strain data after dividing the load by specimen cross-sectional area and deflection by specimen length. For the flexural test, samples were prepared according to ASTM D790-10 using a zwick roell machine. The support span to depth ratio (L/D) was 16 to 1. The maximum stress at failure on the tension side of a flexural specimen was considered the flexural strength of the material. Effects of these mixing methods on the E-glass/epoxy nanocomposites viscoelastic properties was characterized using DMA Q800 from TA Instruments, in the dual cantilever mode, oscillatory frequency of 1 Hz and an amplitude of 15 µm at a heating rate of 5 °C/min in air.

## 4. Conclusions

Proper dispersion of smaller weight percentage of montmorillonite nanoclay particles in E-glass/epoxy composite generated a large surface area for the polymer/filler interaction. This interaction promoted a strong interfacial bond between the fiber and the polymer. The choices of dispersion method influenced to a large extent the degree of exfoliation of the nanoclay particles in the polymer matrix and by extension the fiber/polymer interfacial bond. This study established that significant clay exfoliation was achieved by combining the magnetic stirring and thinky mixing followed by three roll milling to disperse montmorillonite nanoclay in the epoxy matrix. A combination of ultrasound sonication and three roll milling on the other hand, resulted in poor clay exfoliation while the sonication process degraded the polymer network, which adversely affected the nanocomposite final properties relative to the unmodified E-glass/epoxy polymer.
